# Evidence for polarized nanoregions from the domain dynamics in multiferroic LiCuVO_4_

**DOI:** 10.1038/s41598-019-40839-5

**Published:** 2019-03-13

**Authors:** Christoph P. Grams, Severin Kopatz, Daniel Brüning, Sebastian Biesenkamp, Petra Becker, Ladislav Bohatý, Thomas Lorenz, Joachim Hemberger

**Affiliations:** 1 University of Cologne, Institute of Physics II, Zülpicher Str. 77, 50937 Cologne, Germany; 2 University of Cologne, Institute of Geology and Mineralogy, Section Crystallography, Zülpicher Str. 49b, 50674 Cologne, Germany

## Abstract

LiCuVO_4_ is a model system of a 1D spin-1/2 chain that enters a planar spin-spiral ground state below its Néel temperature of 2.4 K due to competing nearest and next nearest neighbor interactions. The spin-spiral state is multiferroic with an electric polarization along the *a* axis which has been proposed to be caused purely by the spin supercurrent mechanism. With external magnetic fields in *c* direction *T*_N_ can be suppressed down to 0 K at 7.4 T. Here we report dynamical measurements of the polarization from *P*(*E*)-hysteresis loops, magnetic field dependent pyro-current and non-linear dielectric spectroscopy as well as thermal expansion and magnetostriction measurements at very low temperatures. The multiferroic transition is accompanied by strong anomalies in the thermal expansion and magnetostriction coefficients and we find slow switching times of electric domain reversal. Both observations suggest a sizable magnetoelastic coupling in LiCuVO_4_. By analyzing the non-linear polarization dynamics we derive domain sizes in the nm range that are probably caused by Li defects.

## Introduction

Multiferroic materials are in the focus of fundamental condensed matter research because of their complex physical properties arising from the coupled magnetic and ferroelectric order parameters^1,2^. Moreover, they also bear a huge potential for technological applications in, e.g., data storage or sensor technologies^3–5^. Of particular importance in this context is a detailed understanding of the dynamics of domain switching processes. The material LiCuVO_4_ originally sparked the scientific interest by showing a broad maximum in temperature dependent magnetic susceptibility^6^, that has subsequently been shown to be caused by 1D spin 1/2 chains. The spin chains are composed of *S* = 1/2 Cu^2+^ ions that are coupled by competing ferromagnetic nearest (*J*_1_ < 0) and antiferromagnetic next nearest neighbor (*J*_2_ > 0) interactions along the crystallographic *b* axis^7^. This frustration leads to the formation of multiferroic cycloidal spin order with a second-order Néel transition at 2.4 K in zero magnetic field^8,9^. Contrary to many other multiferroic compounds, e.g. the manganites^10^, MnWO_4_^11^, or Ni_3_V_2_O_8_^12^, a collinear spin density wave phase above this phase has not been observed. The ferroelectric polarization **P**_**r**_ ∝ **k** × (**S**_**n**_ × **S**_**n**+**1**_) with **k**||*b* is parallel to the *a* axis and has been proposed to be driven purely by the spin supercurrent mechanism^9^.

High magnetic fields induce additional phase transitions^13,14^ at low temperatures. With $H||c$ the electric polarization of the sample is suppressed as the spin structure realizes a collinear spin modulated phase above about 7.5 T. At very high magnetic fields of about 41 T, LiCuVO_4_ enters a spin-nematic phase before the fully spin-saturated phase is reached above 44 T^14,15^.

The goal of our measurements was to study the switchability of the polarization in order to derive both the underlying coupling and domain switching mechanisms. As it turns out, our results call the proposed spin supercurrent mechanism into question by demonstrating the presence of a sizable magnetoelastic coupling and slow dynamics of the ferroelectric domain switching. Furthermore, the low growth dimension and the Vogel-Fulcher-like temperature dependence of the characteristic relaxation time are evidence for a distribution of domain sizes in the nm range determined by structural defects.

## Results

### Crystal growth

**Figure 1:**
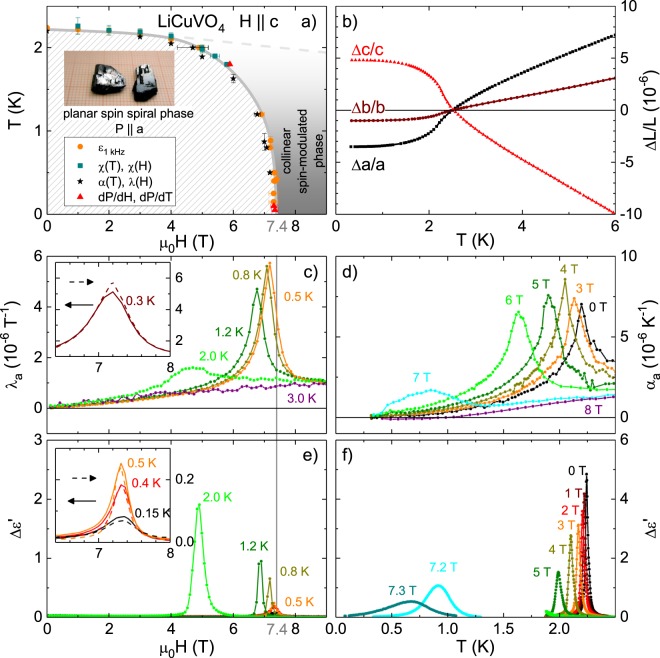
(**a**) Phase diagram of LiCuVO_4_ with $H||c$. (**b**) Relative length change Δ*L*_*i*_/*L*_*i*_ measured parallel to *i* = *a*, *b*, and *c* in *μ*_0_*H* = 0 T. Both the magnetostriction coefficient *λ*_*a*_ in (**c**) and the thermal expansion *α*_*a*_ in (**d**) show the transition into the multiferroic phase as peaks. The phase transition is similarly observed in Δ*ε*′(*H*) in (**e**) and Δ*ε*′(*T*) in (**f**) measured at *ν* =1 kHz. Inserts in (**c**) and (**e**) show enlarged views of the respective low-*T* data measured either with increasing (dashed) or decreasing (solid) magnetic field.

LiCuVO_4_ has an orthorhombically distorted inverse spinel structure of the space group *Imma*^16^ and, on heating, shows peritectic decomposition into CuO and a LiVO_3_-rich melt. For the growth of single crystals there is an access path for compositions LiVO_3_ - LiCuVO_4_ from 10 to 60 mol% LiCuVO_4_^17,18^. We identified a starting composition of 35 mol% LiCuVO_4_, a starting temperature of 910 K and a cooling rate of 0.1 K/h as suitable crystal-growth conditions. Resulting crystals show a platy habit with well-developed morphological face (001) and dimensions up to 15 × 8 × 5 mm^3^ as shown in the inset of Fig. 1(a).

### Phase diagram

Figure 1(a) shows the temperature vs. magnetic-field phase diagram obtained from our measurements for a magnetic field applied along the *c* axis. We restrict ourselves to $H||c$, because for this field direction only the transition from the multiferroic, ferroelectric phase to the paraelectric phase occurs, whereas additional spin-flop transitions are induced for $H||a$ or *b*^19^, which complicate the analysis of the domain dynamics. The multiferroic ordering transition causes significant anomalies in the thermal expansion coefficients *α*_*i*_ measured along all three lattice constants *i* = *a*, *b*, *c*. As is shown in Fig. 1(b), Δ*a*/*a* and Δ*b*/*b* spontaneously contract in the ordered phase and the sum of these contractions is essentially compensated by the expansion of Δ*c*/*c*. Strongly anisotropic strains Δ*L*_*i*_/*L*_*i*_ of comparable magnitudes have also been reported at the multiferroic ordering transitions of MnWO_4_^20^, where the inverse Dzyaloshinskii-Moriya interaction plays an important role in the formation of the multiferroic phase^21^. These strains reveal a pronounced magnetoelastic coupling in LiCuVO_4_, which result from significant uniaxial pressure dependencies of the exchange couplings and also cause the negative thermal expansion of the *c* axis above *T*_N_. In order to derive the phase boundary we measured Δ*L*_*i*_(*H*, *T*) along the *a* axis either as a function of $H||c$ at constant *T* or as a function of *T* at constant *H* and track the pronounced maximums of the magnetostriction *λ*_*a*_ = 1/*L*_*a*_∂Δ*L*_*a*_/*μ*_0_∂*H* and thermal expansion coefficients *α*_*a*_ = 1/*L*_*a*_∂Δ*L*_*a*_/∂*T*, which are shown in Fig. 1(c,d), respectively. This phase transition also causes corresponding peaks in the real part of the dielectric constant *ε*(*T*, *H*) measured at a frequency of 1 kHz, which are displayed in Fig. 1(e,f) after subtracting the background measured in the paraelectric phase, i.e. Δ*ε*′ = *ε*′ − *ε*_∞_, their height is consistent with^19^. The peak positions of the dielectric and expansion measurements are in good agreement, but the temperature- and field-dependent evolution of the peak shapes systematically differ. For example, the peaks of *λ*_*a*_ sharpen on decreasing temperature, whereas those of Δ*ε*′ strongly decrease in magnitude; see panels (c) and (e). Despite this systematic difference, the dielectric and the expansion measurements consistently reveal no indications of hysteresis between the data obtained with increasing or decreasing magnetic field, as is shown in the corresponding insets. This confirms that the nature of this phase transition remains of second order down to the lowest temperature of our measurement with the critical field *μ*_0_*H*_c_ = 7.4 T at 50 mK. Both *T*_N_ and *μ*_0_*H*_c_ from our results are similar to, but slightly lower than, the values of *T*_N_ ≈ 2.5 K and *μ*_0_*H*_c_ ≈ 7.5 T given in^19^.

### Quasi-static polarization

**Figure 2:**
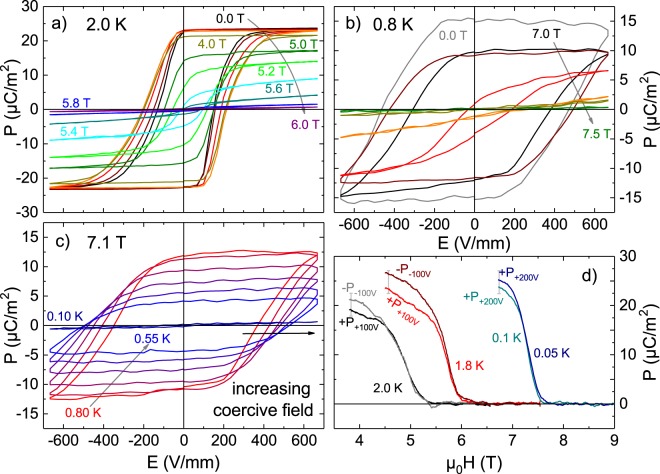
*H*-dependent *P*(*E*) loops at 2.0 K are shown in (**a**) and at 0.8 K in (**b**). (**c**) Shows *T*-dependent *P*(*E*) loops at *μ*_0_*H* = 7.1 T below 0.8 K. Magneto-current measurements in (**d**) show the multiferroic phase transition down to 0.05 K.

To evaluate the switchability of the polarization we measured quasi-static *P*(*E*) hysteresis loops with an effective frequency *ν*_*P*(*E*)_ ≈ 0.01 *Hz* that are shown in Fig. 2. For better visibility the dielectric background *ε*_∞_ ≈ 7.4 was subtracted from all curves. At 2.0 K shown in Fig. 2(a) the hysteresis curves for ${\mu }_{0}H\lesssim 4{\rm{T}}$ are fully formed and reach saturation, similar to measurements already published^22,23^. With further increasing the magnetic field the loops start to close and above the critical field *μ*_0_*H*_c_(2*K*) = 5.8 T the expected paraelectric linear *P*(*E*) is observed.

When cooling the sample to 0.8 K (Fig. 2(b)) it is no longer possible to switch the polarization with the applied electric fields. Here, the hysteresis loops are only fully formed very close to the critical field *μ*_0_*H*_c_(0.8 K) = 7.3 T, demonstrating that the coercive field is higher compared to 2.0 K. This can be seen even better in the temperature dependence of *P*(*E*) loops at 7.1 T in Fig. 2(c). For 0.8 K < *T* < 0.55 K each decrease of the temperature by 0.05 K increases the coercive field and, finally, at 0.1 K the coercive field so strongly exceeds the applied electric field that the *P*(*E*) loop is essentially flat.

To observe the magnetic field dependence of the quasi-static spontaneous polarization also at lower temperatures where the high coercive fields prevent full polarization switching we performed magneto-current measurements. The polarization *P*(*H*), calculated by integration of *I*_mag_(*t*) − *I*_leakage_, is shown in Fig. 2(d) where the spontaneous polarization at the multiferroic phase transition can be seen down to 0.05 K. Within the experimental accuracy the spontaneous polarization below 1.8 K approaches an essentially constant value *P*_S_(*T* → 0) ≈ 30 *μ*C/m^2^.

### Polarization dynamics

As an alternative to the quasi-static “dc” measurements of the polarization described above, we also use sinusoidal electric fields *E*(*t*) = *E*_ac_sin*ωt*. In this case, the polarization *P*(*t*) is obtained by expansion of the applied electric field,

1 $$P(t)={\varepsilon }_{0}{E}_{{\rm{ac}}}\,{\sum }_{n=1}^{\infty }({\varepsilon ^{\prime} }_{n}\,\sin \,n\omega t-{\varepsilon ^{\prime\prime} }_{n}\,\cos \,n\omega t)\mathrm{.}$$

In the experiment, this approach is realized by measuring the higher harmonics of the excitation frequency with the lock-in technique which makes a much broader frequency range up to kHz accessible. For square-type hysteresis loops these measurements in the frequency domain can be used to determine the coercive field *E*_crcv_ and the switchable polarization *P*_sw_ from the lowest-order components *ε*_1_′ and *ε*_1_″ because even terms vanish due to the inversion-symmetry of the ferroelectric hysteresis loops and the magnitude of higher-order terms decreases with 1/*n*. As discussed in detail in^24^ the linear contributions to *ε*_1_′ from phonon modes, *ε*_∞_, have to be removed and both switchable polarization and coercive field can then be calculated with ${P}_{{\rm{sw}}}=\frac{\pi }{4}{\varepsilon }_{0}{E}_{{\rm{ac}}}|{\rm{\Delta }}{\varepsilon }_{1}|$ and *E*_crcv_ = *E*_ac_*ε*″_1_/|Δ*ε*_1_| respectively, where $|{\rm{\Delta }}{\varepsilon }_{1}|={(({\varepsilon ^{\prime} }_{1}-{\varepsilon }_{\infty }{)}^{2}+{\varepsilon }_{1}^{^{\prime\prime} 2})}^{\mathrm{1/2}}$.

**Figure 3:**
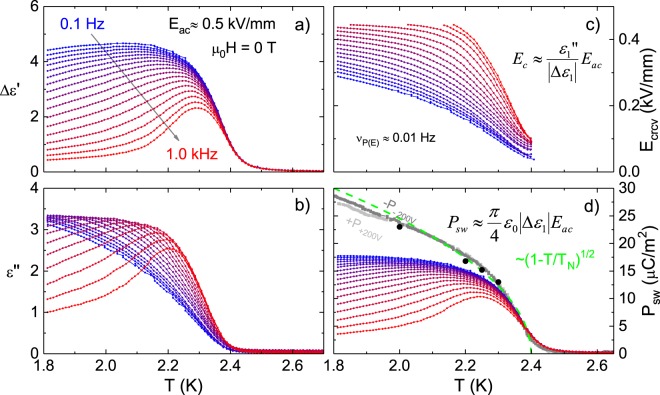
(**a**,**b**) Show measurements of the real and imaginary part of the complex permittivity from 0.1 Hz to 1 kHz at *E*_ac_ = 500 V/mm in zero magnetic field. From this data we calculate *E*_crcv_ in (**c**) and *P*_sw_ in (**d**), the latter is compared to results from quasi-static *P*(*E*) measurements (black) and pyrocurrent (gray). The green line in (**d**) show the *T* dependence of the polarization expected from mean-field theory.

The complex first-order non-linear permittivity ${\varepsilon }_{1}^{\ast }$ was measured in zero magnetic field with *E*_ac_ = 0.5 kV/mm, see Fig. 3(a,b). *ε*_∞_ has been removed from the real part of the permittivity by subtracting the measured results in the paraelectric phase at 3.0 K. In Fig. 3(c) we see the coercive field increasing with decreasing temperature; at the same time this increase is much steeper for higher frequencies where the curves are cut off when *E*_crcv_ reaches about 90% of the applied electric field. The switchable polarization *P*_sw_(*T*, *ν*) is shown in Fig. 3(d). Here, the transition into the ferroelectric phase can be seen in the increase of *P*_sw_ at the Néel temperature *T*_N_ ≈ 2.4 K. While *P*_sw_(*T*, *ν*) increases with decreasing temperature for low frequencies, at high frequencies a maximum appears when the coercive field approaches the applied electric field. For comparison of the absolute values static measurements of the saturation polarization *P*_s_ extracted from pyrocurrent (gray) and *P*(*E*) loops (black) are also shown. Additionally, the dashed line is shown as a guide to the eye

2 $${P}_{{\rm{s}}}\approx 60\frac{\mu {\rm{C}}}{{{\rm{m}}}^{2}}{\mathrm{(1}-T/{T}_{{\rm{N}}})}^{\mathrm{1/2}}$$

where the exponent 1/2 is expected from mean-field theory at a continuous phase transition^25^ and describes the results from the pyrocurrent measurements very well down to *T* ≈ 1.8 K. As the switchable polarization even at lowest frequencies stays clearly below the Landau-type behavior extrapolated from static measurements, part of the sample’s polarization is pinned.

**Figure 4:**
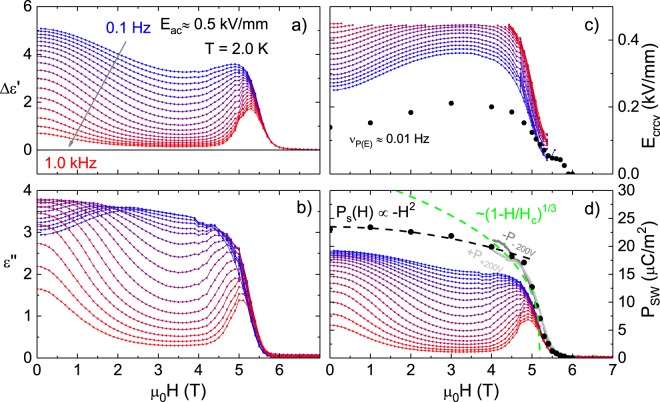
(**a**,**b**) Show measurements of the real and imaginary part of the complex permittivity from 0.1 Hz to 1 kHz at *E*_ac_ = 0.5 kV/mm at 2.0 K. From this data we calculate *E*_crcv_ in (**c**) and *P*_sw_ in **(d**), the latter is compared to results from quasi-static *P*(*E*) measurements (black) and magneto-current (gray). The green line in (**d**) show the expected *H* dependence of the polarization at the phase transition from mean-field theory as well as its −*H*^2^ (black line) dependence in the multiferroic phase.

Similar magnetic-field dependent measurements at 2.0 K are shown in Fig. 4. A key difference to the measurements in zero magnetic field is that with increasing magnetic field the spins are canted out of the *ab*-plane by a magnetic-field dependent canting angle *θ*. Thus, the saturation polarization along the *a* axis also depends on *H* as ${P}_{{\rm{s}}}\propto {\cos }^{2}(\theta )$, i.e. the polarization in the multiferroic phase will be reduced with increasing magnetic field. To quantify the magnetic field dependence further, we use the result from magnetization measurements in *c* direction that show M_c_ ∝ sin(*θ*) ∝ *H* in the multiferroic phase^14^. This leads to an expected field dependence of *P*_s_(*H*) − *P*_s_(0) ∝ *H*^2^ that agrees very well with our results from *P*(*E*) measurements, see Fig. 4(d) (black line). Due to this reduction of *P*_s_ and its interplay with the coercive field we have to distinguish two magnetic-field regimes in our frequency dependent measurements. At *μ*_0_*H* = 0 T we start in the multiferroic phase with frequency-dependent splitting of the curves as discussed above. As the magnetic field is increased to *μ*_0_*H* ≈ 3 T the coercive field increases and, consequently, the switchable polarization is reduced. Here, the depression in the switchable polarization demonstrates that *E*_a*c*_ = 0.5 kV/mm is insufficient to switch the non-pinned polarization fully for frequencies above 0.3 Hz Close to the phase transition at *μ*_0_*H*_c_(2.0 K) ≈ 5.2 T the coercive field drops down and *P*_sw_ increases. Once the coercive field is low enough *P*_sw_(*H*) has a maximum and then follows the saturation polarization that vanishes in the paraelectric phase. In a field dependent measurement also the expected critical exponents at the phase transition differ from the temperature dependent behavior. According to canonical Landau theory, a ferroelectric material with a linear magnetoelectric contribution to the free energy near the multiferroic phase transition obeys ${P}_{{\rm{s}}}\propto {\mathrm{(1}-H/{H}_{{\rm{c}}})}^{{\beta }_{H}}$ with *β*_*H*_ = 1/3^25,26^. The quasi-static results follow this prediction down to *H* ≈ 0.8*H*_c_ while the switchable polarization measured with the dynamical approach follows this prediction only in a much smaller magnetic-field range.

## Discussion

Due to the broad frequency and electric-field range available with the above method we can use the Ishibashi-Orihara model for growth- and nucleation-dominated domain switching^27^ to analyze the underlying mechanism for the switching dynamics of the polarization. This model parametrizes the switching dynamics via an effective domain-growth dimension *d* and an exponent *α* correlating the switching process with the applied electric field. For MnWO_4_, such an analysis reveals a dimensionality ${d}_{{{\rm{MnWO}}}_{4}}=1.8$ and ${\alpha }_{{{\rm{MnWO}}}_{4}}=3.6$^24^. From the magnetic structure of LiCuVO_4_ one may expect $d\gtrsim 1$ reflecting the weakly coupled 1D spin chains. A key prediction of the Ishibashi-Orihara model relates *P*_sw_ to the frequency *ν* and an electric-field dependent factor Φ_*E*_ via

3 $${P}_{{\rm{sw}}}\propto 1-\exp (\,-\,{\nu }^{-d}{{\rm{\Phi }}}_{E}\mathrm{).}$$

**Figure 5:**
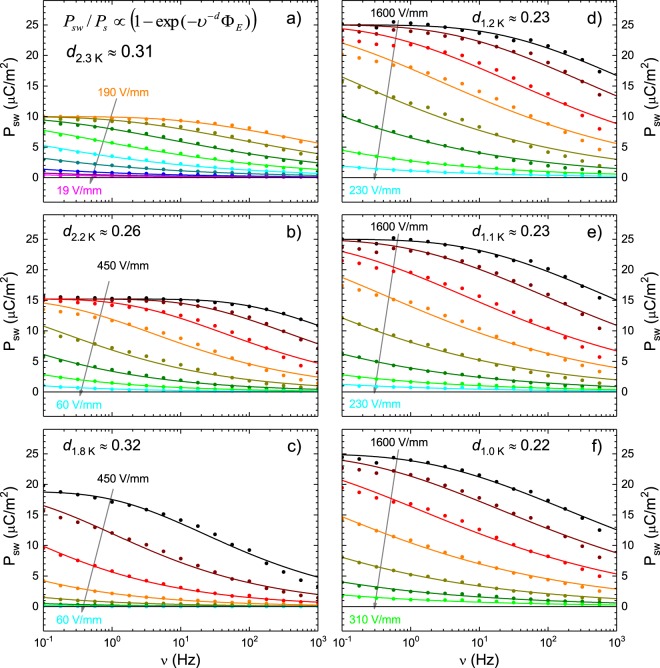
Switchable polarization (symbols) at different temperatures fitted via the Ishibashi-Orihara model (lines) yielding the effective dimensionality *d* and a parameter Φ_*E*_.

We derive *d* and Φ_*E*_ from the corresponding fits of the *P*_sw_(*ν*) data in zero magnetic field at different temperatures. Figure 5 displays the measured *P*_sw_(*ν*) together with fits for *T* = 2.3, 2.2, 1.8, 1.2, 1.1, and 1.0 K. At all temperatures the results are well described by this model and at all temperatures we find $d\ll 1$ with an average value of *d* = 0.26(4). The second parameter in this model, Φ_*E*_, depends on the applied electric field *E*_ac_ and its field dependence can be described by

4 $${{\rm{\Phi }}}_{E}={{\rm{\Phi }}}_{0}\cdot {(\frac{{E}_{{\rm{ac}}}}{{E}_{{\rm{crcv}}}})}^{\alpha }\mathrm{.}$$

**Figure 6:**
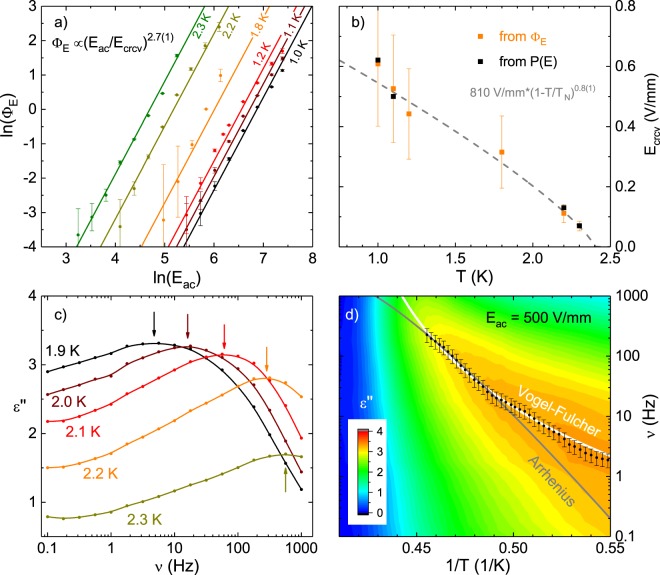
(**a**) The parameters Φ_*E*_ are described by Φ_*E*_ = Φ_0_(*E*_ac_/*E*_crcv_(*T*))^*α*^ with *α* = 2.7(1) for all temperatures. In (**b**) the *T*-dependence of *E*_crcv_ is shown and can be described with *E*_crcv_(*T*) ≈ 810 V/mm⋅(1 − *T*/*T*_*N*_)^0.8^. Exemplary dielectric loss spectra are shown in (**c**), their maximums are marked with arrows. (**d**) *ε*″(*ν*, 1/*T*) with fits to the maximums of the measured dielectric loss spectra (black dots) for Arrhenius-like (gray) and Vogel-Fulcher-like (white) behavior.

As shown in Fig. 6(a) we find *α* = 2.7(1) when fitting all five temperatures simultaneously. The prefactor Φ_0_ ≈ 0.3 follows from a comparison to the coercive field seen in *P*(*E*) loops at different temperatures. In Fig. 6(b) the *T*-dependence of *E*_crcv_ is fitted assuming a power law similar to the saturation polarization, the result is

5 $${E}_{{\rm{crcv}}}(T)\approx 810\frac{{\rm{V}}}{{\rm{mm}}}\cdot {(1-\frac{T}{{T}_{{\rm{N}}}})}^{0.8}\mathrm{.}$$

On the one hand, our data can be almost perfectly parametrized by the model of Ishibashi and Orihara and, therefore, seems to correspond to other multiferroics like e.g. MnWO_4_^24^. On the other hand, however, the outcome of an effective growth dimension $d\ll 1$ suggests, that the switching process is not dominated by the growth of domains^27^.

A “point-like” growth dimension indicates the flipping of stiff domains, a process that induces thermally activated behavior with an energy barrier *U*_B_ ∝ *V*_d_*P*_s_(*T*)*E*_crcv_(*T*) with *V*_d_ denoting the domain volume. Taking into account the above results from equations (2) and (5) we find a temperature dependent energy barrier *U*_B_ as

6 $${U}_{{\rm{B}}}(T)\propto {V}_{{\rm{d}}}{P}_{{\rm{s}}}(T){E}_{{\rm{crcv}}}(T)\approx {V}_{{\rm{d}}}\cdot 49\frac{{\rm{J}}}{{{\rm{m}}}^{3}}\cdot {(1-\frac{T}{{T}_{{\rm{N}}}})}^{1.3}\mathrm{.}$$

In Fig. 6(c) exemplary dielectric loss spectra at five temperatures are shown. At all temperatures the dielectric loss spectra show a maximum at the frequency *ν*_p_ (marked by an arrow in the figure), which corresponds to the relaxation time *τ* = 1/2*πν*_p_ for switching domains of predominant size. Figure 6(d) shows the *T* and *ν* dependence of the dielectric loss *ε*″(*T*, *ν*) as well as *ν*_p_(*T*) from all measured spectra, the error bars mark the spacing in the measured frequencies. This in turn is compared to an Arrhenius-like (gray) and a Vogel-Fulcher-like (white) temperature dependence. The Arrhenius approach *ν*_p_(*T*) ∝ exp(−*U*_B_/*k*_B_*T*) only works close to the phase transition and the increase of *U*_B_ at lower temperatures would increase the slope of the curve bending it further away from the measured data. In contrast, a Vogel-Fulcher-like approach *ν*_p_(*T*) ∝ exp(*U*_B_/(*T* - *T*_VF_)) describes the temperature dependency of the data over a much broader temperature range. The additional parameter in this model, the Vogel-Fulcher temperature *T*_VF_, is found to be *T*_VF_ ≈ *T*_N_. While this type of temperature dependence is typically discussed in the context of glass-like dynamics it has also been used to model the dynamics of polar nanoregions in relaxor ferroelectrics^28^. Despite their different underlying microscopic models both fits yield similar results for the domain volume; *V*_d,A_ ≈ 2.9 · 10^−23^ m^3^ and *V*_d,VF_ ≈ 1.8 · 10^−23^ m^3^ which correspond roughly to spheres with a diameter of 35 nm. This value of course has to be understood as an average over a wide distribution of domain sizes and corresponding switching probabilities as is also suggested by the large widths of the spectra shown in Fig. 5(c).

It has been reported that even carefully prepared LiCuVO_4_ crystals contain a few percent of Li defects^29^. Although the Li in LiCuVO_4_ is nonmagnetic it has been argued that the resulting hole-doped oxygen sites can form singlets with Cu spins that are equivalent to nonmagnetic defects on Cu sites^29,30^. The influence of such defects in LiCuVO_4_ has also been discussed in the context of NMR^31^ and specific heat measurements^32^. As is argued in^32^, non-magnetic defects hardly disturb the formation of the spin-spiral state because of the next-nearest neighbor (NNN) exchange *J*_2_ which still favors the antiparallel alignment of NNN spins that are separated by a nonmagnetic defect. However, such defects are natural sources for antiphase domain boundaries where the spin spiral changes from clockwise to anticlockwise and, accordingly the electric polarization switches from up to down. Concerning the spin-modulated phase, however, non-magnetic defects act as random phase shifts^31^, which severely disturb the formation of long-range incommensurate order and can explain why our thermal expansion data at fields above 7.5 T do not show sizable anomalies that signal a sharp phase boundary between the paramagnetic and the spin-modulated phase. A very similar situation is present in the effective Ising spin-1/2 chain material BaCo_2_V_2_O_8_: large thermal expansion anomalies signal a commensurate spin ordering, whereas a magnetic-field induced incommensurate spin-ordering is almost invisible in thermal expansion^33^.

In summary, by using high quality single crystalline samples we were able to extend the phase diagram of LiCuVO_4_ with $H||c$ down to 0.05 K with measurements of permittivity and magneto-current. Our thermal expansion and magnetostriction data show clear anomalies at the phase transition of the multiferroic phase demonstrating a sizable magnetoelastic coupling. This is compatible with the slow switchability and polarization dynamics from which we conclude that the polarization in LiCuVO_4_ is strongly pinned. Our analysis rules out a domain-growth dominated switching process, instead the polarization dynamics seems to be determined by a distribution of fixed domain sizes in the nm range. We presume that the domain sizes are determined by Li defects that in turn cause the Cu to form nonmagnetic singlets with hole doped oxygen sides. Our data also cast some doubt on the previous conclusion that the polarization in LiCuVO_4_ is purely driven by the spin supercurrent mechanism, because for such a purely electronic mechanism one may expect a comparatively fast polarization dynamics and only a weak magnetoelastic coupling. Comparing our thermal expansion and magnetostriction data to results from MnWO_4_^20^, we find that the relative length changes Δ*L*_*i*_/*L*_*i*_ resulting from the multiferroic ordering are of comparable magnitudes for both materials. Similarly, the observed dielectric relaxation times are also of the same order of magnitude. Thus, we conclude that the inverse Dzyaloshinskii-Moriya interaction cannot be neglected in LiCuVO_4_.

## Methods

For the polarization measurements two contacts where applied with silver paint on opposing ends of the sample along the *a* axis. The resulting capacitive signal was measured with a Novocontrol Alpha-A Analyzer at frequencies up to 1 kHz in high ac electric fields, additional magneto- and pyro-current measurements where performed with a Keithley electrometer 6517B. The dielectric measurements where done in two cryostats, a Quantum Design PPMS and a top-loading dilution refrigerator (Oxford Instruments KELVINOX).

High-resolution measurements of the relative length changes Δ*L*(*T*, *H*)/*L* were performed in a home-built capacitance dilatometer that was attached to a^3^ He system (Oxford Instruments Heliox). The corresponding magnetostriction (*λ*) and thermal expansion (*α*) coefficients were then obtained via numerical differentiation $(\alpha ,\lambda )=\frac{1}{{L}_{0}}\frac{\partial {\rm{\Delta }}L}{\partial (T,{\mu }_{0}H)}$.

## Data Availability

The datasets measured and analysed during the current study are available from the corresponding author on reasonable request.
